# A multifaceted feedback strategy alone does not improve the adherence to organizational guideline-based standards: a cluster randomized trial in intensive care

**DOI:** 10.1186/s13012-015-0285-2

**Published:** 2015-07-08

**Authors:** Maartje L. G. de Vos, Sabine N. van der Veer, Bram Wouterse, Wilco C. Graafmans, Niels Peek, Nicolette F. de Keizer, Kitty J. Jager, Gert P. Westert, Peter H. J. van der Voort

**Affiliations:** Scientific Centre for Transformation in Care and Welfare (Tranzo), University of Tilburg, PO Box 90153, , 5000 LE Tilburg, The Netherlands; Center for Prevention and Health Services Research, National Institute for Public Health and the Environment, PO Box 1, , 3720 BA Bilthoven, The Netherlands; Department of Medical Informatics, Academic Medical Center, PO Box 22660, , 1100 DD Amsterdam, The Netherlands; Center for Public Health Forecasting, National Institute for Public Health and the Environment, PO Box 1,, 3720 BA Bilthoven, The Netherlands; Health Strategy and Health Systems Unit, European Commission, Brussels, Belgium; Scientific Institute for Quality of Healthcare, Radboud University Nijmegen Medical Centre, Nijmegen, The Netherlands; Department of Intensive Care, Onze Lieve Vrouwe Gasthuis, PO Box 95500, , 1090 HM Amsterdam, The Netherlands; TIAS School for Business and Society, Tilburg University, Tilburg, The Netherlands; Health e-Research Centre, The University of Manchester, Manchester, UK

**Keywords:** Multifaceted feedback, Critical care, Quality improvement, Bed occupancy, Nurse-to-patient ratio

## Abstract

**Background:**

Organizational data such as bed occupancy rate and nurse-to-patient ratio are related to clinical outcomes and to the efficient use of intensive care unit (ICU) resources. Standards for these performance indicators are provided in guidelines. We studied the effects of a multifaceted feedback strategy to improve the adherence to these standards.

**Methods:**

In a cluster randomized controlled study design the intervention ICUs received extensive monthly feedback reports, they received outreach visits and initiated a quality improvement team. The control ICUs received limited quarterly feedback reports only. We collected primary data prospectively within the setting of a Dutch national ICU registry over a 14-month study period. The target indicators were bed occupancy rate (aiming at 80 % or below) and nurse-to-patient ratio (aiming at 0.5 or higher). Data were collected per 8-h nursing shift. Logistic regression analysis was performed. For both study end points, the odds ratios (OR) for improvements at follow-up versus at baseline were calculated separately for control and intervention ICUs.

**Results:**

We analyzed data on 67,237 nursing shifts. The bed occupancy rate did not improve in the intervention group compared to baseline (adjusted OR 0.88; 95 % confidence interval (CI), 0.62–1.27) or compared to control group (OR 0.67; 95 % CI 0.39–1.15). The nurse-to-patient ratio did not improve (OR 0.72; 95 % CI 0.41–1.26 compared to baseline and OR 0.65; 95 % CI 0.35–1.19 compared to control group).

**Conclusions:**

A multifaceted feedback intervention did not improve the adherence to guideline-based standards on the organizational issues bed occupancy rate and nurse-to-patient ratio in the ICU. The reasons may be a limited confidence in data quality, the lack of practical tools for improvement, and the relatively short follow-up.

**Trial registration:**

ISRCTN: ISRCTN50542146

## Background

Organizational aspects are associated with clinical outcomes in the intensive care setting. A low nurse-to-patient ratio is associated with unfavorable outcomes [1–5]. In addition, several studies show that a high bed occupancy rate is associated with an increased mortality and also with an increased refusal rate for new appropriately referred patients [6–8]. The bed occupancy rate and the nurse-to-patient ratio are not only relevant clinical issues but also important management tools as they are related to efficiency and costs of intensive care departments [9]. Based on these arguments, national and international societies published guidelines that include recommendations on bed occupancy rates and nurse-to patient ratios [10, 11]. The adherence to these guidelines intends to improve the efficiency and efficacy of intensive care treatment. It is, however, well known that the adherence to guidelines in clinical practice is, in general, limited and improvement in the adherence to guidelines is hard to achieve. Several factors that limit guideline adherence can be distinguished [12]. Performance feedback is a well-established tool to facilitate guideline implementation and to improve the adherence to guideline recommendations. It was shown that feedback reports combined with other strategies as part of a multifaceted intervention were more successful than feedback reports alone [13, 14]. In addition, performance feedback was especially effective in improving the clinical processes of care [14], but data on organizational aspects is lacking. In the intensive care setting, several studies addressed the impact of multifaceted feedback on clinical processes [15–17]. However, no studies concerning multifaceted feedback for organizational standards in the critical care setting are available. Based on these arguments, we conducted a cluster randomized controlled trial (RCT) among Dutch intensive care units (ICUs) to study the effect of a multifaceted feedback intervention on the adherence levels to guideline-based organizational standards, in particular bed occupancy rates and nurse-to-patient ratios.

## Methods

### Context: organizational standards for intensive care in The Netherlands

All Dutch ICUs are closed-format and the majority has an intensivist on call 24 h a day [18]. In 2006, the Dutch Society for Intensive Care (NVIC) developed an evidence-based guideline. This guideline focused on the organizational aspects of intensive care and incorporated several standards [10]. One of these standards is that the bed occupancy rate during a shift should not exceed 80 %. Another standard is that within a shift, no more than two patients should be assigned to one ICU nurse (i.e., a nurse-to-patient ratio of at least 0.5). Both standards were based on evidence that showed crossing these thresholds leads to unfavorable patient outcomes measured as mortality or complication rates.

To ensure that the guideline’s target users, the intensivists, agreed with the standards, the guideline was formally endorsed in a general assembly of the society members. In addition, subsequent to guideline publication, two performance indicators were introduced to monitor adherence to the two standards, one related to bed occupancy and one regarding nurse-to-patient ratio [19]. These indicators were part of a set of 11 structure, process, and outcome indicators [20]. Data of these indicators were collected in the Dutch National Intensive Care Evaluation (NICE) quality registration [21]. ICUs participating in this national registry received a standard quarterly benchmark report on all indicators, including the adherence to these two standards, as a part of the regular service.

### Multifaceted feedback intervention (InFoQi program)

**Fig. 1:**
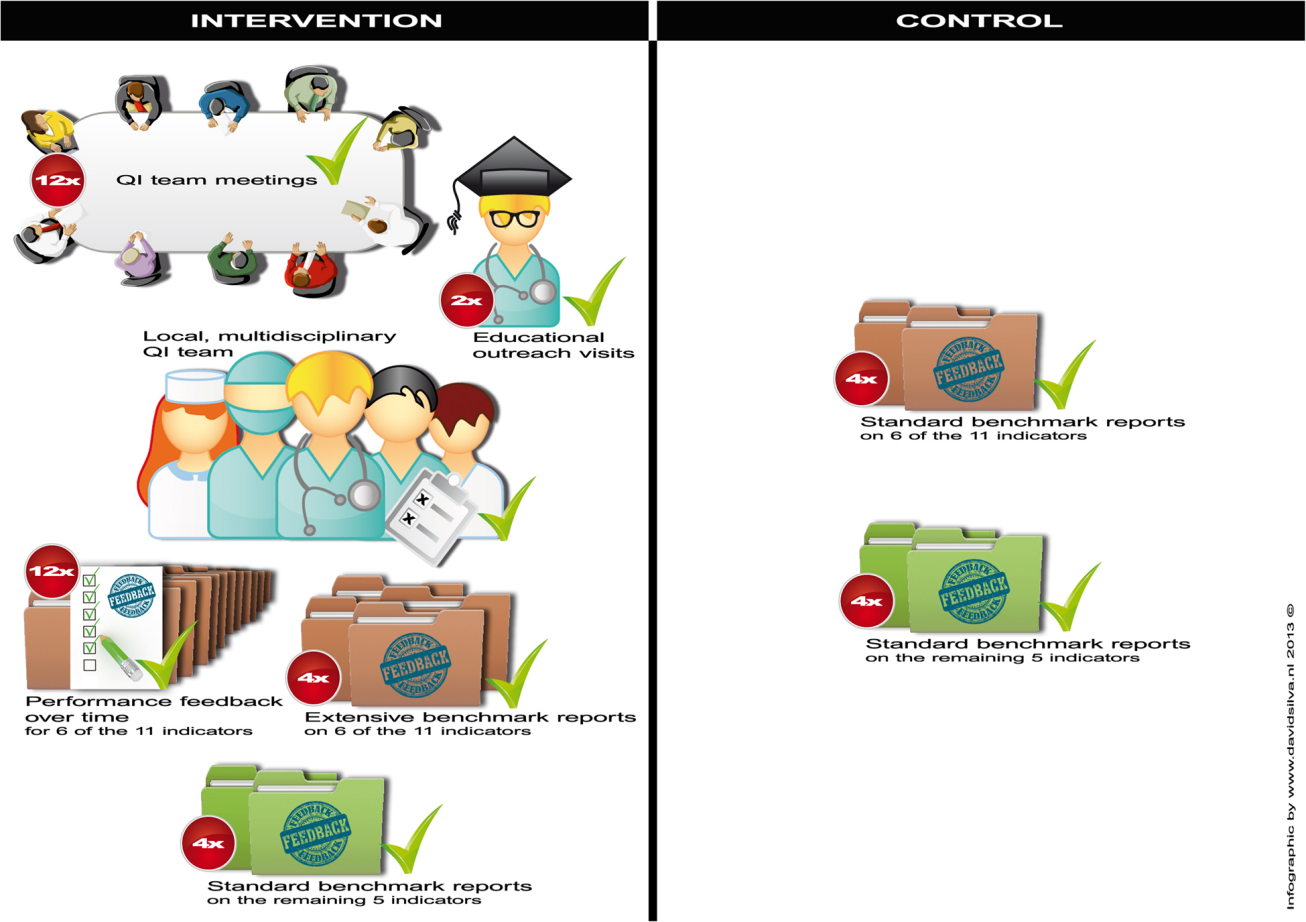
Elements of the multifaceted feedback intervention (InFoQI program) and the quarterly standard feedback reports in the control group

The intervention strategy was an improved NICE feedback reports containing data on 11 indicators. These feedback reports were extended by incorporating the results of a barrier analysis of NICE participants and systematic literature reviews [13, 14, 22]. One of the identified barriers showed a lack of confidence in data quality of the feedback reports. In addition, difficulties in the interpretation of the feedback report hampered quality improvement (QI) activities [22, 23]. The systematic reviews suggested that feedback might be more effective when provided at least monthly in both written and verbal form, when it is combined with the development of a QI plan, and when the feedback has an educational component [13, 23]. Based on this analysis, each intervention ICU during 1 year (1) received monthly and quarterly feedback reports, including information on adherence to the two organizational standards, (2) established a multidisciplinary QI team, (3) received two educational outreach visits. Figure 1 (left side) graphically displays the intervention; a detailed description was published elsewhere [24]. The intervention did not provide explicit tools for professionals to improve bed occupancy rate or nurse-to-patient ratio. The monthly report focused on the ICUs’ own adherence to the standards over time. For example, run charts with occupancy rates per nursing shift three times daily were provided with the 80 % standard visualized as a red horizontal line. The extensive quarterly report focused mainly on comparing an ICU’s adherence level with a benchmark, which was the average adherence level of a group of comparable ICUs. For example, box plots summarizing the nurse-to-patient ratio per week for an individual ICU compared to other ICUs with a similar volume of admitted patients with the guideline-based standard of 0.5 are clearly projected. The monthly reports and the extensive quarterly benchmark reports also contained information on four other clinical indicators next to the two organizational indicators. For the remaining five indicators of the set of 11, no intervention was defined. In each intervention ICU, a multidisciplinary QI team was formed with at least one intensivist, one nurse, and a representative of the ICU management. They had to be available for a minimum of 4 h per month to perform study activities. We suggested adding a data manager as an additional member of the QI team. The teams’ main tasks were to discuss the feedback in a monthly meeting, to formulate a local QI action plan, to initiate and evaluate QI activities, and to share the results with their colleagues. Finally, each ICU in the intervention group received two educational outreach visits by two study investigators (MV and SV). The visits aimed to increase the confidence in data quality, to facilitate correct interpretation of data that was presented in the feedback reports, to translate these data into QI initiatives, and to identify opportunities for improvement.

The ICUs in the control group did not receive any of these interventions except the quarterly standard benchmark reports (Fig. 1, right side).

### Study design

In a clustered RCT, the hypothesis was tested that ICUs receiving the multifaceted feedback intervention improved their adherence to the two organizational standards more than ICUs receiving standard feedback reports only. Dutch ICUs were randomized to either the intervention or the control arm [24]. The Institutional Review Board of the Academic Medical Center (Amsterdam, The Netherlands)—in accordance with Dutch and European legislation—deemed formal IRB approval and patient consent was not necessary because the intervention did not directly involve patient care. The trial was registered at Current Controlled Trials (www.controlled-trials.com; ISRCTN50542146). The study results are reported according the CONSORT statement for cluster randomized trials [25].

### In- and exclusion criteria

ICUs were eligible to participate in the study if they were planning to participate in the Dutch NICE, which included monthly submission of indicator data to the database. ICUs were excluded when they were not able to allocate resources to perform necessary study activities such as establishing a local multidisciplinary QI team. The medical managers of all participating ICUs signed a consent form to formalize leadership support.

### Study endpoints

The primary study endpoints were defined as the following: (1) Adherence to the standard for ICU bed occupancy rate. The ICU bed occupancy rate was defined as the proportion of shifts with a bed occupancy rate 80 % or below (with lower proportions implying higher adherence levels). We calculated bed occupancy rate as the maximum number of patients simultaneously treated on any moment during an 8-h nursing shift divided by the number of operational beds in that same shift. A bed was defined as “operational” when monitoring and ventilation equipment as well as nursing and medical staff is available. (2) Adherence to the nurse-to-patient ratio of 0.5 or above. A higher proportion of nursing shifts with a ratio above 0.5 implied higher adherence levels. The nurse-to-patient ratio was calculated by the maximum number of patients simultaneously present on any moment during an 8-h nursing shift divided by the number of registered qualified ICU nurses in that same shift; student-nurses were not included in this calculation.

### Randomization and blinding

Allocation was based on a stratified randomization with a block size of four. ICUs were stratified by patient volume (more/less than the national median number of ventilated, non-cardiac surgery admissions) and whether they had participated (yes/no) in a pilot study to evaluate feasibility of indicator data collection [19]. An independent researcher—blinded to group assignment—generated the allocation list using a computer-generated randomization scheme. It was impossible to conceal study arm allocation from participating ICUs or from those providing the reports or outreach visits due to the nature of the intervention.

### Data collection

The total study period lasted 16 months starting at randomization to 3 months after sending the last feedback report. These 16 months consisted of a 2-month run-in period, which was the period between randomization and the first outreach visit, directly followed by 14 months of follow-up. The study period for control ICUs was equally defined as a run-in period for the first 2 months after randomization with a follow-up period of 14 months. The data were collected locally by the participating ICUs and uploaded monthly to the national database. The data were commonly recorded per nursing shift using dedicated software. The data included admission and discharge times and dates of individual ICU admissions, the number of operational beds, and the number of qualified nurses per shift. The ICU bed occupancy rates and nurse-to-patient ratios per shift were centrally calculated from the uploaded raw data. The NICE registry assured the quality of submitted data by means of periodical on-site data quality audits and automated data range and consistency checks [21, 26]. Data on QI activities in the intervention ICUs were extracted from the local QI action plans as formulated during outreach visits.

Immediately after inclusion in the study, we interviewed one representative per participating ICU by phone to record whether their unit had any QI infrastructure available (e.g., a quality manager), and how they handled the standard quarterly feedback reports at study baseline. We repeated this interview at the end of follow-up.

### Statistical analyses

The unit of analysis was the individual nursing shift. Each ICU had three nursing shifts per day. The effect of the intervention on the proportion of shifts with a bed occupancy rate below 80 % was analyzed with logistic regression analyses. We used generalized estimation equations with an autoregressive correlation structure to account for clustering of shift occupancy observations within ICUs. The same procedure was followed to analyze the proportion of shifts with a nurse-to-patient ratio above 0.5. The change in proportions was analyzed by testing for the effects of group (intervention versus control), study period (baseline versus follow-up), and the interaction between group and study period.

For both study end points, the odds ratios (OR) for improvements at follow-up versus at baseline were calculated separately for control and intervention ICUs. In addition, the ratio of these ORs for improvement at follow-up (OR intervention/OR control) was calculated [27]. This ratio of ORs should be interpreted as the OR of changes at follow-up in the intervention group adjusted for changes at follow-up in the control group. A ratio of ORs > 1 implied that improvement at follow-up is larger in the intervention group than in the control group.

In all analyses, we adjusted for covariates defined as type of shift (day, evening, and night) and seasonal fluctuation in admission levels by including calendar month dummies for both indicators. In addition, to adjust for differences in organizational structure between the study arms, we used two covariates on the ICU level. In the analyses for the bed occupancy rate, these two covariates were availability of an emergency bed (yes/no) and hospital type (academic or teaching/non-teaching). For the nurse-to-patient ratio, the covariates we added were the proportion of newly admitted mechanical ventilated patients and hospital type.

To account for correlation between the two study endpoints, we performed a secondary analysis in which we added nurse-to-patient ratio as an additional covariate in the analysis for the bed occupancy rate, while also adding bed occupancy rate as a covariate in the analysis for the nurse-to-patient ratio.

### Sample size calculation

To determine the minimally required number of ICUs completing the trial, we used an analysis of the NICE registry data from 18 ICUs in 2008. First, we ranked these 18 ICUs by the percentage of shifts with a bed occupancy rate exceeding 80 % (i.e., failure rate). We than applied the achievable benchmarks of care method to determine what would be an achievable improvement by calculating the difference in failure rate of ICUs in the upper half of the ranking (23 %) and the failure rate among all units (44 %) [28]. This amounted to an absolute reduction of 21 %, corresponding to a relative reduction of 48 %. Calculations based on the Binomial distribution showed that we needed 23 ICUs to detect this difference with 80 % power at a type I error risk (α) of 5 %. In this calculation, we took into account an intra-cluster correlation of 0.28, which we estimated from the 2008 national data. We used SPSS version 16.0 for all statistical analyses.

## Results

### Participating ICUs

**Fig. 2:**
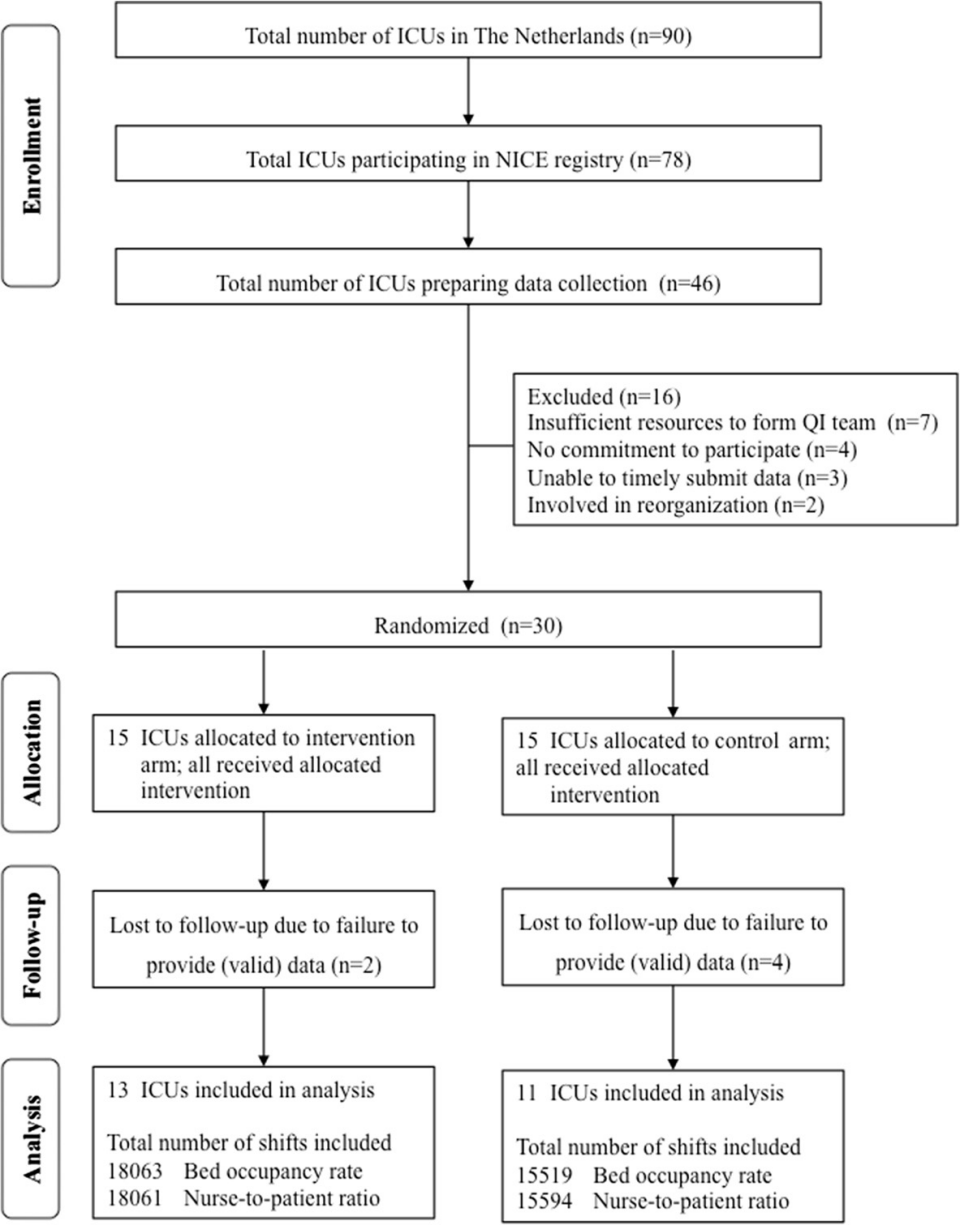
Flow diagram of ICUs and shifts through the trial. *ICU* intensive care unit, *NICE* National Intensive Care Evaluation, *QI* quality improvement

From the 78 ICUs that submitted data to the national database, 46 ICUs were preparing to collect and submit the organizational indicators concerning bed occupancy and nurse-to-patient ratio. Thirty of these 46 ICUs provided consent to participate in the study. Fifteen units were assigned to the intervention arm and 15 to the control arm (Fig. 2). The ICUs were included consecutively between January 2009 and November 2009.

Two ICUs from the intervention group were excluded from analysis and four from the control group because they failed to provide valid data throughout the study period. Finally, 24 ICUs were included in the analyses, with a total of 33,582 nursing shifts for bed occupancy and 33,655 shifts for nurse-to-patient ratio. Table 1 presents the baseline characteristics of the included ICUs and shifts.

**Table 1:** Baseline characteristics of participating ICUs and included shifts in the InFoQI program

	Intervention	Control
ICU level characteristics		
Number included in analysis	13	11
Academic or teaching hospital	9	5
Cardiac surgery center	2	2
Emergency bed available	8	8
Participated in indicator pilot study	5	3
Shift level characteristics		
Number included in analysis		
Bed occupancy rate	18,063	15,519
Nurse-to-patient ratio	18,061	15,594
Number of operational beds^a^	13.6 (8.1)	12.98 (7.7)
Bed occupancy rate^a^	75.6 (2.0)	79.5 (2.2)
Percentage of shifts adhering to standard for bed occupancy rate^b^	50 %	43 %
Number of qualified nurses^a^	7.1 (4.5)	6.6 (5.5)
Nurse-to-patient ratio^a^	0.72 (0.3)	0.69 (0.3)
Percentage of shifts adhering to standard for nurse-to-patient ratio	76 %	74 %

*ICU* intensive care unit, *QI* quality improvement

^a^Values are mean (standard deviation) of day, evening, and night shifts together

^b^Expressed as the proportion of shifts with bed occupancy rate below 80 %

^c^Expressed as the proportion of shifts with nurse-to-patient ratio above 0.5

### Effect of the multifaceted feedback intervention

Over the study period, the multifaceted feedback intervention showed no effect in the study end points compared to standard feedback reports alone. Table 2 shows the decline in the proportion of shifts with a bed occupancy rate below 80 % compared to baseline for intervention compared to control ICUs (OR, 0.88; 95 % confidence interval (CI), 0.62–1.27).

**Table 2:** Results of the multifaceted intervention program on study end points

	Crude outcome^a^		Crude effect of intervention compared to baseline^b^		Adjusted^c^ effect of intervention compared to baseline		Crude effect of intervention compared to control group^d^		Adjusted^c,d^ effect of intervention compared to control group	
Organizational indicators	Intervention (%)	Control (%)	Main effect (95% CI)	*P* value	Main effect (95% CI)	*P* value	Main effect (95% CI)	*P* value	Main effect (95% CI)	*P* value
Proportion of shifts with bed occupancy rate below 80 %	48	51	0.94 (0.68–1.29)	0.69	0.88 (0.62–1.27)	0.50	0.68 (0.41–1.13)	0.14	0.67 (0.39–1.15)	0.14
Proportion of shifts with nurse-to-patient ratio above 0.5	70	75	0.75 (0.44–1.30)	0.30	0.72 (0.41–1.26)	0.24	0.70 (0.38–1.28)	0.25	0.65 (0.35–1.19)	0.17

*CI* confidence interval

^a^Percentages of shifts in follow-up that adhere to the guideline recommendations for each indicators

^b^Odds ratio for improvement in follow-up period for the intervention group compared to baseline period

^c^Bed occupancy rate adjusted for type of shift, month of the year, availability emergency bed, and academic/teaching or non-teaching unit. Nurse-to-patient ratio adjusted for type of shift, month of the year, ratio of newly admitted mechanical ventilated patients in a shift and academic/teaching or non-teaching unit

^d^Ratio of odds ratios for improvement in follow-up between groups, calculated as odds ratio of the intervention divided by the odds ratio of the control group

The proportion of shifts with a bed occupancy rate below 80 % is higher in follow-up compared to baseline for the control ICUs, but the ratio of change during follow-up between intervention and control ICUs was not statistically significant (ratio of ORs, 0.67; 95 % CI, 0.39–1.15).

The proportion of shifts with a nurse-to-patient ratio below 0.5 showed a non-significant reduction during follow-up compared to baseline for intervention ICUs (OR, 0.72; 95 % CI, 0.41–1.26). The ratio of change in intervention versus control ICUs was not statistically significant (ratio of ORs, 0.65; 95 % CI, 0.35–1.19).

The secondary analyses in which the additional covariates were added did not change the final conclusion.

### Quality improvement activities

All intervention ICUs established a QI team and received two educational outreach visits. Variation in the number of initiated QI actions during the outreach visits varied between ICUs, ranging from zero to four actions aiming to improve bed occupancy rate, and zero to ten actions related to increasing nurse-to-patient ratios. Table 3 shows examples of QI actions; most QI actions investigated the quality of the data and the low adherence to the guideline standards, rather than changes in the processes of resource planning.

**Table 3:** Type and examples of planned QI actions as formulated during outreach visits and the number of QI plans (*n* = 13) in which they appeared

Type of action	Bed occupancy	Nurse-to-patient ratio
Understanding low or high variation in performance	12	11
	Verifying unusually high or low occupancy rates in specific shifts	Develop standard registration procedure for combined intensive care—recovery nursing shifts^a^
Investigate low performance	9	8
	Investigate effect of daily operating-room schedule on bed occupancy rate	Investigate the number of student-nurses in shifts with a ratio below the lower threshold^b^
Adjust (process of) resource planning	2	4
	Introduce labeling of the “best patient” in each shift who could be discharged in case of an emergency admission	Take on additional part-time nurses or share nursing capacity with medium care to increase nursing team’s versatility

^a^Some hospitals had combined ICU-recovery nursing shifts, which may interfere with the registration of nurse-to-patient ratio

^b^Student-nurses are not taken into account for the nurse-to-patient ratio

During the study period in the control group, two ICUs established a local QI team, while in one ICU, this was discontinued. In addition, four control ICUs reported to have started using the standard quarterly benchmark reports.

## Discussion

This study shows that the intervention with extended feedback reports, QI teams, and educational outreach visits did not lead to a significant improvement in the adherence to guideline-based standards for bed occupancy rates and nurse-to-patient ratios in the Dutch intensive care setting compared to standard feedback reports alone. To our knowledge, this is the first study that evaluated the effect of a multifaceted feedback intervention on the adherence to organizational guidelines in the critical care setting. Our findings are in contrast with improvements shown in adherence to clinical guideline standards after implementation of a multifaceted feedback approach [15–17]. Apparently, organizational improvements are more difficult to achieve than clinical improvements. This discrepancy may have several reasons such as study design, difficulties in changing organizational indicators, and the type of intervention.

### Study design

The lack of effect may in part be explained by weaknesses in the study design. First, the control group also received feedback on adherence levels, which diluted the contrast between the two study arms. This lack of contrast could not be avoided because all ICUs in our study already participated in the national registry with regular performance feedback in some form. Another limitation is that we had to exclude six of the ICUs from our analysis due to absent or incomplete data, which may have limited the power of the study. This relatively large drop out may be due to the time-consuming and manual recording of operational beds and qualified nurses (three times daily) in ICUs without a patient data management system. However, the power analysis indicates that sufficient ICUs in our analyses remained.

Strength of our study is the use of a cluster randomized design. In addition, we investigated the potential achievable improvement prior to the study for both study end points by using actual data and a validated method [28].

### Organizational versus clinical indicators

It is shown that the bed occupancy rate and nurse-to-patient ratio are difficult to change. These organizational issues are difficult to influence by nature, as they need change in the number of admitted patients or the number of nurses. Changing the admission rate may be difficult because a large proportion of admissions are unplanned. In order to increase the number of nurses, it is necessary to search for new employees in a short supply market or to achieve flexible availability of nursing staff. In addition, to find money for this investigation, most ICUs are hampered by budgetary cuts in health care. It is also true that the two endpoints may co-interfere as an increasing number of nurses may increase the number of operational beds (providing that the unit can increase its beds and apparatus) and thereby improve both bed occupancy rate and nurse-to-patient ratio.

### Intervention and quality initiatives

The multifaceted intervention did not include practical tools to achieve improvements in the study endpoints. These practical implications had to be designed by the QI teams their selves. These tools might, for instance, have been extended information on patient admission and discharge logistics or tools to achieve flexible availability of nurses. However, our data show that most of the initiatives from the QI teams aimed to “increase the confidence in data,” which does not directly target the adherence to the guideline standards. This finding is in line with those of Bradley et al. [29]. They described that efforts in QI were most effective when physicians perceived that the data were credible, and this data credibility often took time to develop in a hospital [29]. In our prospectively performed barrier analysis, we found that the confidence in data was a barrier that had to be solved before this study could be able to achieve an improvement. Indeed, the process evaluation of our study did show an increase in confidence in indicator data quality [30], but the participating ICUs were apparently stuck in improving data quality and were not able to take a step beyond this issue. This finding suggests that the 14-month time frame of this study was too short to achieve a measurable effect. This is confirmed by the finding that the increase in confidence in the data took several months to occur. Consecutive initiatives after data improvement were, however, not often undertaken. In this respect, it is unknown whether the intensivists and nursing staff had enough latitude to change the relevant processes. In addition to the relatively short follow-up, as stated above, the feedback of organizational data may also be more difficult to explain. It may be true that recipients need more background information to correctly interpret data on their organizational performance [31]. The organizational indicators used in this study are based on evidence-based standards, but conflicting interests may have influenced the interpretation of the data and the initiation of QI activities. Economic targets and quality improvement targets may not be inline. For example, bed occupancy rates over 80 % can be economically beneficial but can have negative effects for the accessibility of intensive care facilities. Finally, another explanation for the observed outcomes could be that the ICUs in the intervention group showed better results at baseline compared to the control group, which results in limited room for improvement in the follow-up period or due to regression to the mean.

### Extrapolation and future steps

From this study, it can be learned that an improvement in organizational aspects of a guideline in the intensive care setting is not easy to achieve in a 12-month episode. For organizational indicators, in contrast to clinical indicators, a distinct implementation approach might be necessary and more time might be needed. Although our study has been conducted within the domain of intensive care, our conclusions might be true in other settings as well. Also, it is unknown whether our results are true for other countries. Future studies are needed to gain insight on these questions.

## Conclusions

Bed occupancy rate and nurse-to-patient ratio are important hallmarks for an efficient use of critical care resources and they are associated with clinical outcomes. Improvement of the adherence to guideline-based standards on these organizational issues in critical care was studied using a cluster randomized trial. A multifaceted feedback intervention using extensive feedback data, a local quality improvement team, and an educational outreach team appeared to be ineffective. The ICUs appeared to have limited confidence in the correctness of the feedback data. The ICUs appeared to be unable to take appropriate steps beyond improvement of data quality. Moreover, this study suggests that efforts to improve bed occupancy rate and nurse-to-patient ratio may require an intervention with practical tools for improvement and a longer follow-up.
